# Role of Catalase in Oxidative Stress- and Age-Associated Degenerative Diseases

**DOI:** 10.1155/2019/9613090

**Published:** 2019-11-11

**Authors:** Ankita Nandi, Liang-Jun Yan, Chandan Kumar Jana, Nilanjana Das

**Affiliations:** ^1^Department of Biotechnology, Visva-Bharati University, Santiniketan, West Bengal 731235, India; ^2^Department of Pharmaceutical Sciences, UNT System College of Pharmacy, University of North Texas Health Science Center, Fort Worth, TX 76107, USA; ^3^Department of Chemistry, Purash-Kanpur Haridas Nandi Mahavidyalaya, P.O. Kanpur, Howrah, West Bengal 711410, India

## Abstract

Reactive species produced in the cell during normal cellular metabolism can chemically react with cellular biomolecules such as nucleic acids, proteins, and lipids, thereby causing their oxidative modifications leading to alterations in their compositions and potential damage to their cellular activities. Fortunately, cells have evolved several antioxidant defense mechanisms (as metabolites, vitamins, and enzymes) to neutralize or mitigate the harmful effect of reactive species and/or their byproducts. Any perturbation in the balance in the level of antioxidants and the reactive species results in a physiological condition called “oxidative stress.” A catalase is one of the crucial antioxidant enzymes that mitigates oxidative stress to a considerable extent by destroying cellular hydrogen peroxide to produce water and oxygen. Deficiency or malfunction of catalase is postulated to be related to the pathogenesis of many age-associated degenerative diseases like diabetes mellitus, hypertension, anemia, vitiligo, Alzheimer's disease, Parkinson's disease, bipolar disorder, cancer, and schizophrenia. Therefore, efforts are being undertaken in many laboratories to explore its use as a potential drug for the treatment of such diseases. This paper describes the direct and indirect involvement of deficiency and/or modification of catalase in the pathogenesis of some important diseases such as diabetes mellitus, Alzheimer's disease, Parkinson's disease, vitiligo, and acatalasemia. Details on the efforts exploring the potential treatment of these diseases using a catalase as a protein therapeutic agent have also been described.

## 1. Introduction

Reactive species (RS) are highly active moieties, some of which are direct oxidants, and some have oxygen or oxygen-like electronegative elements produced within the cell during cellular metabolism or under pathological conditions. Some of the reactive species are free radicals such as the hydroxyl radical and the superoxide radical, and some are nonradicals such as hydrogen peroxide. Free radicals are any independent species which consist of one or more unpaired electrons in their atomic or molecular orbital. They are generally unstable, short lived, but usually chemically reactive. They can react with any molecule either by oxidizing it or by causing any other kind of chemical modification. Free radicals can potentially oxidize all cellular biomolecules including nucleic acids, proteins, and lipids. For example, peroxidation of omega-6 polyunsaturated fatty acid (such as arachidonic acid and linoleic acid) leads to the production of 4-hydroxynonenal (HNE), which is one of the main reactive aldehydes produced by oxidative stress [1]. There are many reactive species and free radicals [2] which are listed in Table 1.

These free radicals are formed in the cell during normal cellular metabolism as mitochondrial electron transport chain, *β*-oxidation of fatty acids, and cytochrome P450-mediated reactions and by the respiratory burst during immune defense. For example, autooxidation of some biologically important substances such as FADH_2_ and tetrahydropteridines can yield O_2_^**·**–^ in the presence of oxygen [3]. The imbalance between production and quenching of these reactive substances through antioxidant mechanisms causes oxidative stress. The loss of functionality and adaptability of important biomolecules due to oxidative stress are two interdependent biological processes, which are among the important factors that mediate aging. The free radical hypothesis, also known as oxidative stress hypothesis, is one of the strongly supported theories which can define the causes behind the aging process.

Oxidative stress has been implicated in many metabolic and neurologic degenerative disorders. Degenerative diseases, where the function and structure of a tissue or organs deteriorate over time such as in Alzheimer's disease, Parkinson's disease, diabetes, cataracts, cancer, and cardiovascular disease, have been attributed to oxidative stress conditions and the process of natural aging. Thus, oxidative stress, aging, and degenerative diseases are interconnected.

**Figure 1:**
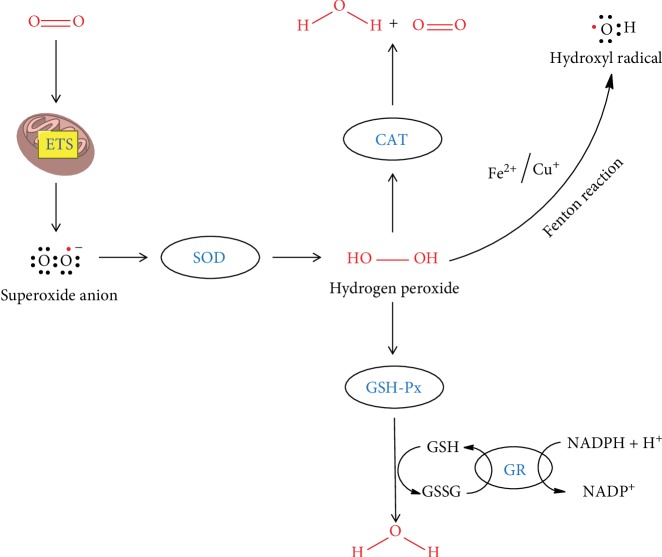
Relationship between catalase and other antioxidant enzymes.

**Figure 2:**
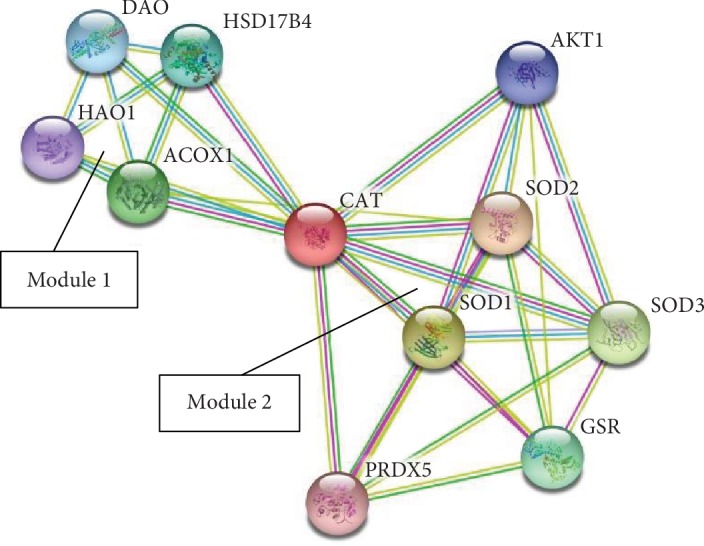
STRING analysis of interaction of catalase with other proteins.

The body has a defense mechanism against oxidative stress in which both enzymatic and nonenzymatic molecules are the two prime components. This antioxidant defense system consists of some enzymes, some proteins, and a few low molecular weight molecules. The antioxidant enzymes can catalytically remove the reactive species. For example, superoxide dismutase dismutates superoxide into hydrogen peroxide which is in turn degraded by catalase or by glutathione peroxidase. The relationship between the different antioxidant enzymes is depicted graphically in Figure 1. Transferrin, metallothionein, and caeruloplasmin are some of the proteins which can reduce the availability of prooxidants such as transition metal ions like iron ions and copper ions which can produce a hydroxyl radical from hydrogen peroxide by the Fenton reaction. The low molecular weight antioxidants include ascorbic acid, *α*-tocopherol, glutathione, and uric acid, which neutralize the RS by scavenging the whole molecule or its byproducts, by reducing it by or participating in any form of chemical reaction leading to complete or partial destruction of it or its byproducts. The interaction of catalase with other antioxidants and proteins can be predicted by the STRING (Search Tool for the Retrieval of Interacting Genes/Proteins) analysis [4, 5]. STRING is a biological database used to study protein-protein interaction. The STRING network analysis of catalase's interaction with other proteins has been categorized into two distinct modules (Figure 2). Module 1 contains four proteins which are basically involved in the pathways of peroxisomes including CAT and three proteins of module 2 such as SOD1 (superoxide dismutase 1), SOD2 (superoxide dismutase 2), and PRDX5 (peroxiredoxin 5) [6–8] (Supplementary [Supplementary-material supplementary-material-1]). In module 1, ACOX1 (peroxisomal acyl coenzyme A oxidase), HSD17B4 (peroxisomal multifunctional enzyme), and HAO1 (hydroxyacid oxidase 1) are involved in the fatty acid oxidation pathway in the peroxisome while the protein DAO (D amino acid oxidase) is involved in the amino acid metabolism pathway in the peroxisome [6–8] (Supplementary [Supplementary-material supplementary-material-1]). All the components of module 1 are involved in different metabolic pathways. The proteins in module 2 are mainly involved in responses against oxidative stress. All the proteins have antioxidant activity except AKT1 (RAC-alpha serine-threonine protein kinase). AKT1 is a serine-threonine protein kinase which is involved in cell survival, metabolism, growth, and angiogenesis. All the proteins of both modules 1 and 2 including CAT have catalytic activity and are located in the lumen of intracellular organelles. SOD2 and AKT1 of module 2 including CAT were involved in the longevity regulating pathway and FOXO signaling pathway in mammals [6–8] (Supplementary Figures [Supplementary-material supplementary-material-1] and [Supplementary-material supplementary-material-1]). But in multiple other species, SOD1 and SOD3 (superoxide dismutase 3) were also involved along with SOD2, AKT1, and CAT [6–8] (Supplementary [Supplementary-material supplementary-material-1]). Among the reactive species, hydrogen peroxide is freely diffusible and is relatively long-lived. It acts as a weak oxidizing as well as reducing agent; however, it is not very reactive, but it is the progenitor of many other reactive oxygen species (ROS). It has been demonstrated to oxidatively modify glyceraldehyde-3-phosphate dehydrogenase by oxidation of the labile essential thiol groups at the active site of this enzyme [2]. In most cellular injuries, this molecule is known to play an indirect role. One of the most important products is the formation of a more reactive free radical ^·^OH radical in the presence of transition metal ions such as Fe^2+^ by means of the Fenton reaction.

There are many enzymes that are able to neutralize hydrogen peroxide. These enzymes include catalase, glutathione peroxidase, and other peroxidases such as cytochrome c peroxidase and NADH peroxidase [2]. Catalase is a key enzyme which uses hydrogen peroxide, a nonradical ROS, as its substrate. This enzyme is responsible for neutralization through decomposition of hydrogen peroxide, thereby maintaining an optimum level of the molecule in the cell which is also essential for cellular signaling processes. The importance of the enzyme can be gauged from the fact of its direct and indirect involvement in many diseases and infections. In this review, an attempt has been made to correlate the role of catalase with the pathogenesis and progression of oxidative stress-related diseases. A brief account of catalase, its isoforms, structure, and reaction mechanism, and its relation with some common important disorders is described in this review article.

## 2. Catalase

**Figure 3:**
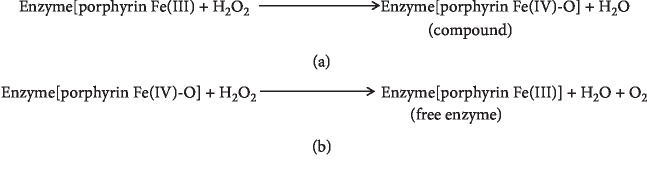
Steps in catalase reaction: (a) first step; (b) second step.

A catalase (E.C. 1.11.1.6) is one of the most important antioxidant enzymes. It is present in almost all aerobic organisms. Catalase breaks down two hydrogen peroxide molecules into one molecule of oxygen [9] and two molecules of water in a two-step reaction [10]. The same is represented in Figure 3 as derived from Ivancich et al. [11] and Lardinois [12]. The first step of the reaction mechanism involves formation of a spectroscopically distinct intermediate compound I (Figure 3(a)) which is a covalent oxyferryl species (Fe^IV^O) having a porphyrin *π*-cation radical, through the reduction of one hydrogen peroxide molecule [11]. In the second step reaction (Figure 3(b)), compound I is reduced through redox reactions by a two-electron transfer from an electron donor (the second molecule of hydrogen peroxide) to produce the free enzyme, oxygen, and water [10].

In 1937, the protein was first crystallized from bovine liver at Sumner and Dounce's laboratory [13]. The first prokaryotic catalase was purified from an aerobic bacterium, *Micrococcus lysodeikticus*, in 1948 [14]. The gene coding for catalase is the *CAT* gene which is positioned in chromosome 11 in humans. In the following decades, several studies have been carried out on prokaryotic catalase and also on the lower eukaryotic catalase. In particular, research on catalase from *Saccharomyces cerevisiae* has generated data and information on the evolution of the enzyme at the molecular level. It has also been reported that catalase is an important enzyme implicated in mutagenesis and inflammation conditions as well as during the suppression of apoptosis [15–18] which are all known to be associated with oxidative stress conditions.

Catalase has been characterized from many eukaryotic as well as prokaryotic organisms.  Table 2 summarizes some basic physiochemical information available in the literature to date on catalase from different organisms. Based on the differences in their sequence and structure, there are three different types of catalase. The monofunctional heme-containing enzyme is the most widespread one. It is present in all aerobic organisms. The bifunctional catalase-peroxidase belongs to the second class, which is relatively less abundant in nature. This enzyme also contains a heme group. It is closely related to the plant peroxidases with structural and sequence similarities. The third class belongs to the Mn-containing catalase group which lacks the heme group.

**Figure 4:**
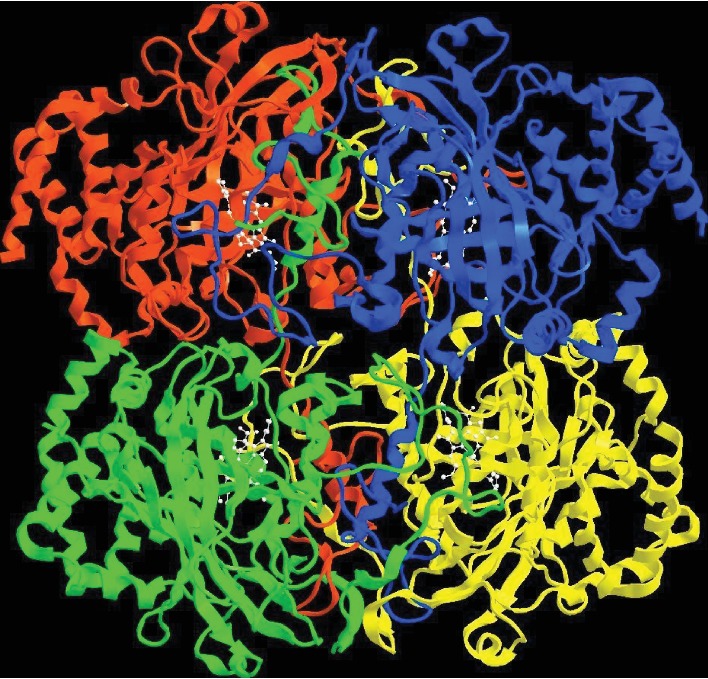
Crystal structure of human erythrocytic catalase [20] PDB ID: 1F4J.

Humans possess a typical monofunctional heme-containing catalase having a prosthetic group of ferric protoporphyrin IX which reacts with hydrogen peroxide. Located in the peroxisomes, the enzyme has a molecular mass of approximately 220-240 kDa [19]. It is a tetrameric protein with each subunit divided into four domains, the N-terminal threading arm, C-terminal helices, wrapping loop, and *β* barrel [20] (Figure 4). Each subunit has a hydrophobic core comprising eight stranded *β* barrels surrounded by *α*-helices. These *β* barrels are antiparallel with each other. The heme distal side of the subunit is made up of the first four *β* strands (*β*1-*β*4) of the *β* barrel domain and the remaining four strands (*β*5-*β*8) play a part in the NADPH binding pocket. The N-terminal threading arm of a subunit (residues 5-70) intricately connects two subunits by hooking through a long wrapping loop (residues 380-438). Finally, a helical domain at one face of the *β* barrel is composed of four C-terminal helices. Tetramerization forces the N-terminal threading arms from the arm-exchanged dimer to cover the heme active site for the other pair of dimers and suggests that catalase fits the more general pattern of domain swapping with the arm-exchange being a later, tetramer-dependent elaboration. Throughout the protein, water fills in packing defects between the four domains of the subunit and between subunits within the tetramer. Only the hydrophobic *β* barrel and the immediate vicinity of the active site are substantially devoid of these structural water molecules. From XRD studies, the root mean square deviation (r.m.s.d.) coordinate differences between the four subunits were found to be 0.156 Å for the backbone atoms, 0.400 Å for the side chains, and 0.125 Å for the heme groups [21]. The 3D structure of the enzyme at 1.5 Å was elucidated in 2001 [22]. The crystal structures of human catalase show that the active site iron is pentacoordinated. The negatively charged heme carboxylate radical forms salt bridges to three arginine residues (Arg72, Arg117, and Arg365) which likely aid in heme burial and help increase the redox potential of the compound I porphyrin radical and are conserved in bacterial, fungal, plant, and animal catalase. Besides the heme group, the active conformation of the enzyme consists of one tightly bound NADPH molecule in each subunit. There are various reports on the role of this NADPH molecule. It has been demonstrated to obstruct the formation of Fe (IV)oxo-ligated porphyrin, an inactive form of catalase—by hydrogen peroxide, and to also slowly induce the removal of inactive catalase [19, 23].

## 3. Catalase-Related Diseases

**Figure 5:**
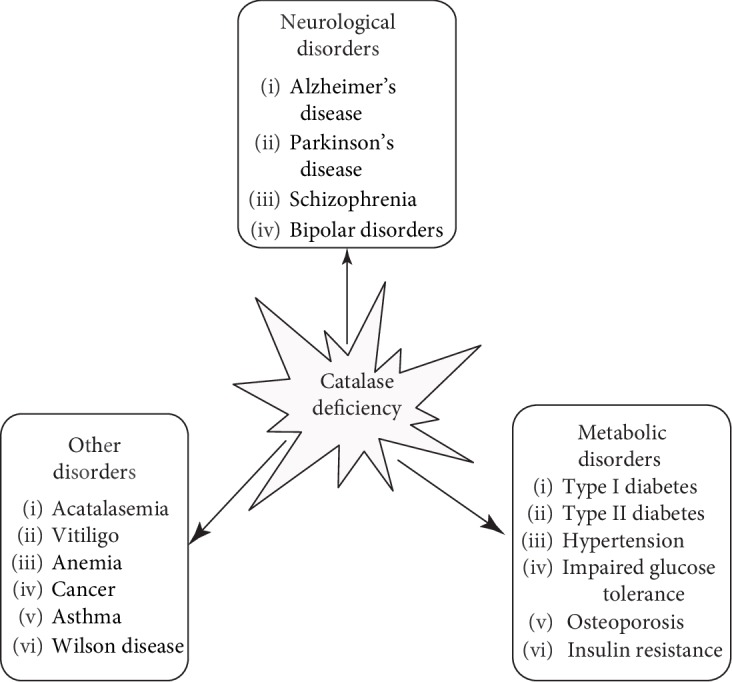
List of some diseases linked to catalase deficiency.

Catalase deficiency or malfunctioning is associated with many diseases such as diabetes mellitus, vitiligo, cardiovascular diseases, Wilson disease, hypertension, anemia, some dermatological disorders, Alzheimer's disease, bipolar disorder, and schizophrenia [24–26] (Figure 5). It has been reported that an anomaly of catalase activity is inherited in acatalasemia which is a rare genetic disorder (also known as Takahara disease) [27]. It is an autosomal recessive trait and characterized by a reduced level of catalase. Catalase has a prime role in regulating the cellular level of hydrogen peroxide [28, 29], and its hydrogen peroxide catabolism protects the cells from oxidative assault, for example, by securing the pancreatic *β* cells from hydrogen peroxide injury [30, 31]. Low catalase activities have been reported in schizophrenic patients such as also in patients with atherosclerosis [32].

**Figure 6:**
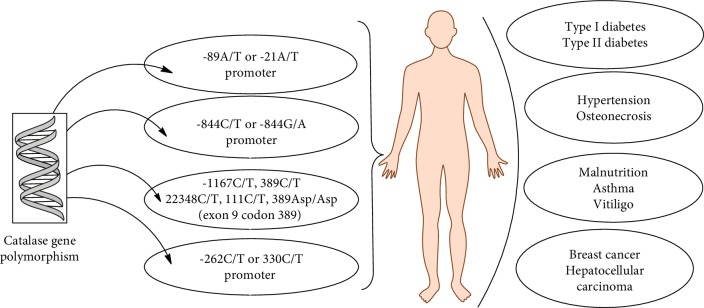
Association of catalase polymorphism with risk of some widespread diseases.

Genetic variations in the catalase gene and in its promoter region also play a role in the pathogenesis of various diseases which is depicted in Figure 6. Several studies have investigated *CAT* polymorphism and its involvement in the development of various diseases as well as its role as regulator in the *CAT* gene expression. Single nucleotide polymorphisms of the *CAT* gene in the promoter region possibly affect the transcription frequencies resulting in low *CAT* expression [33, 34]. The most common polymorphisms that influence the transcription of the *CAT* gene and also affect catalase activity are -262C/T and -844G/A or -844C/T in the promoter region [35]. There are many other polymorphisms involved in the development of numerous diseases which varies amongst populations. *CAT* -262C/T polymorphism is related to type 1 diabetes and breast cancer [36–38]. Two single nucleotide polymorphisms of *CAT* gene, viz., 1167T/C and -262C/T, have been reported to have a strong association with type 1 diabetes mellitus [36]. The functional consequence of this 1167T/C polymorphic positioned in exon 9 is not known. But in case of -262C/T, the variation shows significant functional significance. It influences the AP-2 and Sp-1 (nuclear transcriptional factors) binding and also effects the expression as well as the level of catalase in the red blood cells [36]. In Swedish populations, the concentration of erythrocytic catalase in individuals carrying the TT genotype was high compared to those of the CC genotype [39]. In Russian populations—on the other hand—the individuals carrying the CC genotype have a higher risk of developing type 1 diabetes than those carrying the TT genotype [36]. The blood catalase level was found to be low in CC individuals which results in oxidative stress conditions, thereby promoting type 1 diabetes [36]. Another single nucleotide polymorphism of the *CAT* gene 111C/T in exon 9 was examined among different forms of diabetes and showed a very poor association [40]. The *CAT* -262C/T polymorphism has an association with breast cancer. The CC genotype showed higher catalase activity in red blood cell as compared to TT and TC genotypes with a correlated reduced risk of breast cancer by 17% [38]. However, it must be noted here that this population study was performed with a much lesser number of individuals. Studies have shown that the level of -262C/T polymorphism effects not only the transcriptional activity but also the level of catalase in red blood cells [37]. Another common *CAT* polymorphism is -844C/T or -844G/A which might result in a lower catalase level by influencing the transcription frequency. *CAT* -844C/T polymorphism has a strong association with hypertension among the Chinese population [41]. Hypertension is a multifactorial complex lifestyle disease. Among Japanese populations, this -844C/T polymorphism has been reported to show a strong association with hypertension [42]. But the functional relationship is not very clear. The *CAT* -844G/A, -89A/T, and -20T/C polymorphisms have been shown to be associated with malnutrition [43]. This polymorphism might affect the transcription rates thereby lowering the catalase level. The -89A/T polymorphism has also been reported to exhibit an association with vitiligo and osteonecrosis [44, 45]. The variant with *CAT* -89A/T has been reported to be associated with a significantly reduced level of catalase with a correlation with developing vitiligo in the Chinese population [44]. The *CAT* 389C/T genotype has no reported association with vitiligo in the Chinese population, but a connection has been established in North America and the United Kingdom [44, 46, 47]. The relation of these genotypes with vitiligo pathogenesis is discussed in a later section. The *CAT* -89A/T, -20T/C, +3033C/T, +14539A/T, +22348C/T, and +24413T/C polymorphisms might be involved in osteonecrosis amongst Korean populations [45]. Data from all the studies show different polymorphisms of the *CAT* gene among different populations in various regions of the world. Further population-based research across the world is required to gain a clear idea about the association of the *CAT* gene in different diseases. In the future, this might unlock new therapeutic approaches by regulating the *CAT* gene.

### 3.1. Diabetes Mellitus

Diabetes mellitus is a common disease nowadays. They are caused by a bundle of metabolic disorders, distinguished by high levels of glucose in the blood due to improper secretion of insulin or its activity or both. It can lead to other secondary afflictions such as nerve damage, blindness, heart disease, stroke, and kidney disease. There has been a significant rise in the diabetes-affected population in recent years. It is estimated that, worldwide, the number of diabetes-affected adults will increase more than twofold from the 135 million affected in 1995 to approximately 300 million by 2025 [48] and 629 million by 2045 [49], and the majority of increment will be from developing countries such as India [48].

**Figure 7:**
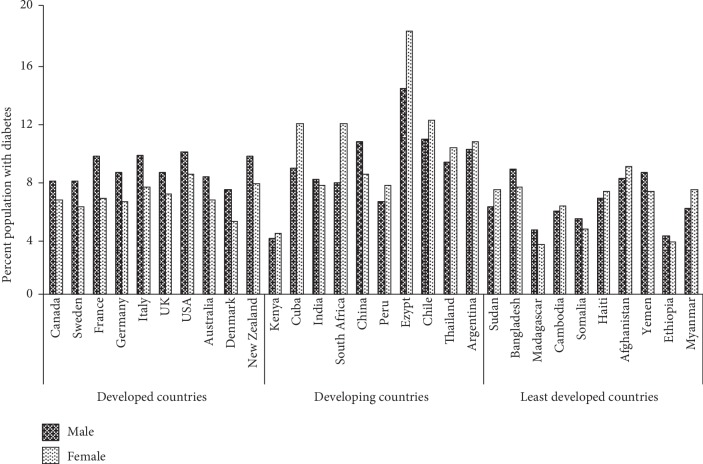
Prevalence of diabetes amongst males and females in some countries in 2018 (data source: World Health Organization-Diabetes Country Profile 2018).

The 2018 data from the diabetes country profile from the World Health Organization (WHO) [50] is depicted in Figure 7 which shows the prevalence of diabetes amongst both genders in different countries classified according to their economic status by a United Nation's report. The disease seems more prevalent in the developed nations, and the percentage of the affected population seems to show a more or less uniform level in all these countries. A lot of discrepancies in the level are observed amongst the developing nations with the highest percentage of the population being affected in Egypt. Less prevalence is observed amongst the least developing nations indicating that lifestyle and diet play a major role in development of the disease as observed amongst the developed nations. Gender also seems to play a role with more prevalence of the disease among the females than the males in the developing nations indicating that societal norms may also play a role. In contrast, males seem more susceptible in developed nations, which indicates a possible genetic and lifestyle role in development of the disease.

There are two general forms of diabetes mellitus, type 1 and type 2. Type 1 diabetes mellitus is a juvenile form and insulin-dependent diabetes which accounts for approximately 10% of all cases, but it may also develop in adults [51]. In this case, pancreatic *β* cells are destroyed by autoantibodies rendering the cells incapable of producing insulin. This autoimmune disease has a correlation between immunologic and genetic factors. There are three major types of autoantibodies found in type 1 diabetes such as GDP65, IA2, and insulin autoantibodies, but antibodies against insulin can be identified mostly in young patients and may be lacking in adults [52, 53]. These antibodies bind mainly to the conformational epitopes on the B chain of insulin. The genetic feature shows a relationship between type 1 diabetes and some alleles of the HLA complex. There is a strong connection between the progression of type 1 diabetes and the presence of HLA class II alleles.

Type 2 diabetes mellitus is the most common form of the disease, accounting for approximately 90% of all diabetes cases. It occurs primarily due to low production of insulin and secondarily also due to insulin resistance by the body's cells. The *β* cells of islets of Langerhans become damaged which make them unable to produce insulin. Oxidative stress has been demonstrated to be an important factor responsible for the advancement of type 2 diabetes. It has been demonstrated that hydrogen peroxide acts as an oxidant and damages the *β* cell interrupting the signaling pathway of insulin production [30, 54, 55]. According to a study from Prof. Kassab's laboratory, a four-fold increase in the concentration of hydrogen peroxide was observed in type 2 diabetes mellitus patients than in the healthy controls [56]. This observation was corroborated with observations of low catalase activity in the *β* cells in hyperglycemic mice models [57].

Another form of diabetes known as pancreatogenic diabetes has been classified as type 3c diabetes mellitus (T3cDM). T3cDM is the result of pancreatitis (both acute and chronic), cystic fibrosis in the tissue of pancreas, inflammation, and damage of pancreatic tissue [58, 59]. The damage of exocrine pancreatic peptide (PP) and pancreatic enzymes occurs at the early phase of pancreatic diabetes. The reduction of the glycogen level due to damage of *α* cells occurs at a late phase of pancreatic diabetes. The resultant elevated level of glucagon can lead to hyperglycemia in diabetes mellitus [60]. There are many aspects associated with the pathophysiology of pancreatic diabetes. Immunopathogenesis is one of the important aspects which contribute to the development of pancreatic diabetes. Different proinflammatory cytokines like tumor necrosis factor *α*, interferon *γ*, and interleukin 1*β* are involved in the pathogenesis of pancreatic diabetes [60]. Higher concentration of cytokines leads to the dysfunction of the *β* cells at an early stage of chronic pancreatitis [61]. At higher concentration, interleukin 1*β* induces the apoptosis of *β* cells by the NF*κ*B pathway [62]. Higher concentration of interferon *γ* diminishes the translocation of pancreatic and duodenal home box 1 (PDX1), a transcription factor. PDX1 is important for the development of pancreatic cells through maturation of *β* cells and also via duodenal differentiation [63]. Reduction of survivability and differentiation of *β* cells occur in patients with chronic pancreatitis due to loss of PDX1. Hydrogen peroxide plays a central role in this pathway as a signaling molecule [64]. At lower concentration, hydrogen peroxide plays as a signaling molecule while it becomes toxic at higher concentration [65] and catalase plays an important role in maintaining homeostasis of the cells by degrading hydrogen peroxide. The activity of catalase in the serum was observed to be high in acute pancreatitis [66] and persists at its elevated level for as long as 10 to 14 days [66]. Therefore, the high catalase activity may contribute to the pathogenesis of T3cDM in an indirect way by maintaining the hydrogen peroxide concentration which would induce the synthesis of proinflammatory cytokines resulting in pancreatic diabetes.

Gestational diabetes mellitus (GDM) is another common form of diabetes among pregnant women. The pathogenesis of GDM is very similar to type 2 diabetes mellitus. There are several factors including ethnicity, maternal age, hypertension, obesity, and polycystic ovary syndrome (PCOS) which are associated with the possibility of developing GDM [67, 68]. Pregnant women with GDM have higher risk of developing type 2 diabetes mellitus after pregnancy [68]. The offspring of gestational diabetic mothers are prone to development of different diseases like hypertension, different metabolic syndrome, and chronic kidney disease [69, 70]. These birth defects might be due to higher concentration of reactive oxygen species and lowering of the antioxidant defense which in turn make the cell more susceptible to oxidative insults [70, 71]. GDM usually develops in the second and third trimesters of the pregnancy period. Reports on the link of catalase with GDM are very conflicting. It has been reported that oxidative stress is high in the second and third trimesters of pregnancy and the catalase activity was also low during this period [72, 73]. The blood catalase activity has been reported to be low in pregnant women with GDM compared to nonpregnant and pregnant nondiabetic healthy control women [72]. However, the blood catalase activity was observed to increase in the third trimester than in the second trimester in pregnant individuals with GDM [72]. In another study, low blood catalase activity has been observed in pregnant women with GDM [40]. As already mentioned, there is poor association between 111C/T polymorphism and different forms of diabetes mellitus which include GDM [40]. The mRNA expression of the *CAT* gene in the placenta of gestational diabetic pregnant women was found to be higher in comparison to that in normal pregnant women [74]. So it may be concluded from the above that catalase might have a relation with GDM pathophysiology during pregnancy, but further research to establish the facts is needed.

Hydrogen peroxide has been implicated to act as a cellular messenger in the signaling pathway for insulin secretion by inactivating tyrosine phosphatase [65, 75–78]. It has been postulated that catalase in the liver may confer cellular protection by degrading the hydrogen peroxide to water and oxygen [28–31]. Lack of catalase can contribute to the development of diabetes mellitus [76, 79] with a positive correlation being observed between diabetes mellitus in acatalasemic patients. It is estimated that approximately 12.7% of acatalasemic/hypocatalesemic patients are also affected by diabetes mellitus [79]. It was proposed that catalase deficiency may be responsible for the development of diabetes mellitus in an indirect way [24]. The *β* cells are known to be oxidant sensitive. These cells are not only deprived of catalase but also have a higher concentration of mitochondria [80] which is one of the major sources of superoxide and hydrogen peroxide in the cell through the electron transport pathway. Therefore, in acatalasemic/hypocatalesemic patients, a low amount of oxidative stress over a long period of time may result in the accumulation of oxidative damage in the *β* cells that results in the onset of diabetes [76, 79].

There are many vascular complications in diabetes mellitus including microvascular complications (diabetic retinopathy, nephropathy, neuropathy, etc.) and cardiovascular complications [81]. Oxidation plays an important role in different complications which occur in both type 1 and type II diabetes. Due to the low expression levels or activity of catalase, the concentration of hydrogen peroxide may increase in the cells creating oxidative stress conditions causing the progression of different types of complications. In the case of diabetes retinopathy, the retina is damaged by retina neovascularization where new vessel origination from existing veins extends to the retinal inner cells [82] leading to blindness [83]. Vascular endothelial growth factor (VEGF) is a prime inducer of angiogenesis, a procedure of new vessel development. Nox4, a major isoform of NADPH oxidase, is predominant in the endothelial cells of the retina. It causes the generation of hydrogen peroxide instead of other reactive species [84]. Hydrogen peroxide may have a role as a signaling molecule in the VEGF signaling molecule. An upregulation of the Nox4 expression with downregulation of the catalase expression and/or activity in diabetes increases the hydrogen peroxide concentration which promotes retinal neovascularization through the VEGF signaling pathway [82]. In a study on a diabetic rat model, high concentration of hydrogen peroxide was observed in the retinal cells, creating oxidative stress conditions within the cell [85]. Since retinal cells have high content of polyunsaturated fatty acid content [86], they can be oxidized by the hydroxyl radicals generated from hydrogen peroxide by the Fenton reaction. High levels of lipid peroxides and oxidative DNA damage have been observed in diabetic retinopathy [87–90].

In a recent study, researchers have been able to distinguish five distinct clusters of diabetes by combining parameters such as insulin resistance, insulin secretion, and blood sugar level measurements with age of onset of illness [91]. Group 1 essentially corresponds to type 1 diabetes while type 2 diabetes is further subdivided into four subgroups labelled as group 2 to group 5. Individuals with impaired insulin secretion and moderate insulin resistance are labelled under group 2 (the severe insulin-deficient diabetes group) while in group 3, the severe insulin-resistant diabetes patients with obesity and severe insulin resistance are included. Group 4 is composed of the mild obesity-related diabetes patients who are obese and fall ill at a relatively young age while the largest group of patients is in group 5 with mild age-related diabetes in mostly elderly patients. A relationship between this new classification of diabetes with catalase expression levels or its activity has still not been probed for a link, if any, and needs further research.

### 3.2. Neurological Disorders

#### 3.2.1. Alzheimer's Disease

Alzheimer's disease is one of the onset of dementia diseases in adults [92]. According to the report of the Alzheimer's Association, approximately 5.5 million people in the United States of America were suffering from Alzheimer's disease in 2017. It is estimated that by 2050, the prevalence of Alzheimer's diseases will increase immensely from 4.7 million in 2010 to an estimated 13.8 million in 2050 [93].

Many factors including smoking and diabetes are associated with a higher risk of dementia. Alzheimer's disease is characterized by deposition of senile plaques of amyloid *β* peptides in the brain [94, 95]. There are several studies which demonstrate that amyloid *β* peptides are toxic to neurons in culture [96–109]. Amyloid *β*, an amyloid precursor protein processing (APP) product, is a soluble component of the plasma and cerebrospinal fluid (CSF). In all cases of Alzheimer's disease, it has been observed that the soluble amyloid *β* is converted to insoluble fibrils in senile plaques through formation of protein-protein adducts [96–99, 101].

It has been observed using *in vitro* cell culture studies that the nascent amyloid *β* is nontoxic but aged amyloid *β* becomes toxic to neurons [110]. It has been observed that amyloid *β* peptide is responsible for hydrogen peroxide accumulation within the cultures of neuroblastoma and hippocampal neurons [111, 112] probably by the direct binding of amyloid *β* to catalase leading to decreased enzyme activities [26]. These findings led to development of the hypothesis that the catalase-amyloid *β* interaction may play a significant role in the increment of hydrogen peroxide in the cells linking the accretion of amyloid *β* and development of oxidative stress conditions in Alzheimer's disease [26]. So the current hypothesis regarding the mechanism of amyloid *β*-stimulated oxidative damage in cells is that amyloid *β* directly interacts with catalase by binding with the protein and deactivating its catalytic activity thereby creating oxidative stress conditions. In addition, full-length amyloid *β* peptides bind to Cu^2+^ at their N-terminal section of the peptide and reduce it to the Cu^+^ form [113]. It has been reported that amyloid *β*-Cu^+^ complex can lead to hydrogen peroxide production [114, 115]. Therefore, catalase has both a direct and an indirect relationship with the pathogenesis of Alzheimer's disease.

#### 3.2.2. Parkinson's Disease

Parkinson's disease is an age-associated neurological disorder with the initial symptom as a simple tremor of the hand which gradually affects the whole body movement diminishing the quality of life severely with the advancement of the disease. Its clinical manifestations include bradykinesia, rigidity, resting tremor, and postural instability. It starts with rhythmic tremor of limbs especially during periods of rest or sleep. At the developing stage of the disease, patients face difficulties in controlling movement and muscle rigidity. Due to this muscular rigidity, slowness of movement and slowness of initiation of movement occur.

The disease is characterized by the exhaustion of dopamine due to damage of dopamine-producing neurons in the substantia nigra pars compacta (SNpc) [116–118]. It has been demonstrated that Parkinson's disease-affected patients suffer from 100-200 SNpc neuronal damages per day [119]. As various factors such as genetic inheritance, environmental toxins, oxidative stress, and mitochondrial dysfunction are probably involved in the pathogenesis of the disease, it is very challenging to understand the pathogenesis of Parkinson's disease.

It has been demonstrated that a protein, alpha (*α*) synuclein, is closely related to the cytopathology and histopathology of Parkinson's disease [120]. It has been observed that mutation in a gene responsible for the production of *α*-synuclein results in the production of a mutant protein that can promote the deposition of dopamine in the cytoplasm of neurons [121]. The small neurotransmitter molecules like dopamine are synthesized in the cytoplasm and are transferred to small vesicles as it becomes oxidized at the physiological pH. Mutant *α*-synuclein permeabilizes these vesicles causing leakage of the dopamine into the cytoplasm where it autooxidizes producing hydrogen peroxide, superoxide molecules, and toxic dopamine-quinone species creating oxidative stress conditions [122]. Mutant *α*-synuclein protein is also known to inhibit the expression and activity of catalase [123]. Arrest of catalase activity by *α*-synuclein is probably by hindering the peroxisome proliferator-activated receptor *γ* (PPAR*γ*) transcription activity, which regulates the *CAT* gene expression [123]. Based on such experiments, it may be concluded that the low catalase activity and high hydrogen peroxide production in Parkinson's disease might be due to (the indirect) inhibition of catalase expression by the *α*-synuclein molecule.

### 3.3. Vitiligo

Vitiligo is one of the chronic pigmentary disorders where skin melanocyte cells—the pigment responsible for the color of the skin—are damaged or are unable to produce melanin. Various studies have shown that the catalase levels in the epidermis of vitiligo patients are lower as compared to those of the healthy control subjects [124, 125] with a resultant increase in the concentration of hydrogen peroxide. In the cell, hydroxyl radicals can be produced spontaneously from hydrogen peroxide through photochemical reduction, i.e., the Haber-Weiss reaction [15]. These hydroxyl radicals are able to oxidize lipids in the cell membrane. This may be the cause behind damage of keratinocytes and melanocytes in the epidermal layer of the skin in such patients [126–130]. Moreover, the inhibitory effect of hydrogen peroxide or allelic modification of the *CAT* gene results in low catalase activity. However, it has been observed that there is an erratic relationship between catalase polymorphism and vitiligo. The 389C/T polymorphisms of exon 9, codon 389, and -89A/T of the promoter region were studied in vitiligo patients [34, 44, 46, 47, 131, 132]. But the results were not observed to be consistent. Amongst the Chinese population, an association was observed in AT and TT genotypes with the increased risk of vitiligo whereas no association was observed between vitiligo and the -89A/T *CAT* polymorphism in the Korean population [34, 44]. In the case of 389C/T polymorphism, several studies showed no difference between the controls and vitiligo patients [34, 44, 46, 131] although contrary results have also been obtained in a few studies [47, 131]. It has been reported that a mutation in the *CAT* gene might change the gene expression and/or cause structural changes in the keratinocytes and/or melanocytes [46]. Though the results are inconsistent from population studies, an interconnection between the pathogenesis and catalase may still be possible as scattered demonstrations are reported in the literature. Therefore, further studies to understand the link is necessary.

### 3.4. Acatalasemia

Acatalasemia (AC) is a hereditary disorder which is linked with the anomaly of catalase enzyme affecting its activity. In 1948, Takahara, a Japanese otolaryngologist, first reported this disorder [133, 134]. He found that four out of seven races in Japan had the same genetic flaw [135]. His *ex vivo* experiments consisted of filling the mouth ulcer of a diseased patient with hydrogen peroxide. Since no bubble formation was observed, he concluded that a catalase or its enzymatic activity is absent in the saliva of the patients. In honor of his primary findings, this disease was christened as the Takahara disease. Acatalasemia and hypocatalasemia signify homozygotes and heterozygotes, respectively. The heterozygote of acatalasemia shows half of the catalase activity than normal and this phenotype is known as hypocatalasemia [136]. Depending on the geographical location from where it has been first studied, there are different types of acatalasemia described as Japanese, Swiss, Hungarian, German, and Peruvian types. Approximately 113 acatalasemic patients have been reported to date from all over the world.

Two kinds of mutations in the catalase gene have been reported to be involved in the Japanese acatalasemia. A splicing mutation has been held responsible for Japanese acatalasemia I where a substitution of a guanine residue with adenine residue at position 5 of intron 4 disturbed the splicing pattern of the RNA product producing a defective protein [137]. In Japanese acatalasemia II, a frame shift mutation occurs due to the deletion of thymine in position 358 of exon 4 which modifies the amino acid sequence and produces a new TGA (stop) codon at the 3′ terminal. Translation of this mutated strand produces a polypeptide of 133 amino acid residues. This is a truncated protein that is unstable and nonfunctional [138].

Aebi et al. first described Swiss acatalasemia [139–141]. The study on the fibroblast from Swiss acatalasemia patients suggests that structural mutations in the *CAT* gene are responsible for inactivation of catalase [142]. Goth, a Hungarian biochemist, first described Hungarian acatalasemia in 1992 after studying the disease in two Hungarian sisters. He found that the catalase activities in the blood of these two acatalasemic sisters were 4.4% and 6.7% of the reference catalase activity in the healthy population whereas the level of activity in hypocatalasemic patients was 38.9% [24]. Studies at his laboratory led Goth to suggest that mutations of the *CAT* gene and resultant structural changes in the catalase protein are responsible for Hungarian acatalasemia. This laboratory also reported that there was a risk of diabetes mellitus amongst the Hungarian acatalasemic family members though further biochemical and genetic analysis needs to be performed to validate the hypothesis that acatalasemic patients have more chance of developing diabetes mellitus [79]. There are generally four types of Hungarian acatalasemia which varies according to the (different) site of gene mutation in the DNA. The same is represented in Table 3.

## 4. Therapeutic Role of Catalase

Catalase is one of the most important antioxidant enzymes. As it decomposes hydrogen peroxide to innocuous products such as water and oxygen, catalase is used against numerous oxidative stress-related diseases as a therapeutic agent. The difficulty in application remains in delivering the catalase enzyme to the appropriate site in adequate amounts. Poly(lactic co-glycolic acid) nanoparticles have been used for delivering catalase to human neuronal cells, and the protection by these catalase-loaded nanoparticles against oxidative stress was evaluated [143]. It was observed that the efficiency of the encapsulation of catalase was very high with approximately 99% enzymatic activity of encapsulated catalase along with significantly sustained activity over a month. The nanoparticle-loaded catalase showed significant positive effect on neuronal cells preexposed to hydrogen peroxide reducing the hydrogen peroxide-mediated protein oxidation, DNA damage, mitochondrial membrane transition pore opening, and loss of membrane integrity. Thus, the study suggests that nanoparticle-loaded catalase may be used as a therapeutic agent in oxidative stress-related neurological diseases [143]. Similar research has been conducted using EUK 134 which is a class of synthetic superoxide dismutase/catalase mimetic as an effective therapeutic agent in stroke [144]. EUK 134 is a salen-manganese complex which has both high catalase and superoxide dismutase activity. It was concluded from these studies on the rat stroke model that EUK 134 may play a protective role in management of this disease.

Studies using Tat-CAT and 9Arg-CAT fusion proteins as therapeutic agents have also been carried out with encouraging results [145]. To study the effect of these fusion proteins under oxidative stress conditions, mammalian cell lines (HeLa, PC12) were transduced with purified fusion Tat-CAT and 9Arg-CAT protein and these cells were exposed to hydrogen peroxide. It was found that the viability of the transduced cells increased significantly. It was also observed that when the Tat-CAT and 9Arg-CAT fusion proteins were sprayed over animal skin, it could penetrate the epidermis and dermis layers of the skin. The fusion proteins transduced in mammalian cells were active enzymatically for over 60 h after which they became unstable. This study suggests that these fusion proteins can be potentially used as protein therapeutic agents in catalase-related disorders [145].

Amyotrophic lateral sclerosis (ALS) is one of the most common types of progressive and fatal neurological disorders which results in loss of motor neurons mostly in the spinal cord and also to some extent in the motor cortex and brain stem. Amongst the two distinct types of ALS, the familial form (FALS) accounts for 10% of all ALS cases and 15 to 20% of FALS cases are related to the SOD1 gene mutation, an antioxidant enzyme which scavenges the superoxide radical. In some of the FALS cases, it has been found that the mutation in the SOD1 gene is not linked to a lowered activity of SOD1. Rather, the mutated SOD1 has toxic properties with no lowering of the enzymatic activity. This mutated SOD1 protein reacts with some anomalous substrates such as hydrogen peroxide using it as a substrate and produces the most reactive hydroxyl radical which can severely damage important biomolecules [146]. Mutated SOD1 also has the potential to use peroxynitrite as an atypical substrate leading to the formation of 3-nitrotyrosine which results in the conversion of a functional protein into a nonfunctional one [147]. Catalase can reduce the hydrogen peroxide concentration by detoxifying it. Therapeutic approaches using putrescine-modified catalase in the treatment of FALS have also been attempted [148]. It was found that putrescine-catalase—a polyamine-modified catalase—delayed the progression of weakness in the FALS transgenic mouse model [148]. Thus, the delay in development of clinical weakness in FALS transgenic mice makes the putrescine-modified catalase a good candidate as a therapeutic agent in diseases linked with catalase anomaly. In this connection, it must be mentioned that the putrescine-modified catalase has been reported to exhibit an augmented blood-brain barrier permeability property while maintaining its activity comparable to that of native catalase with intact delivery to the central nervous system after parenteral administration [148]. Therefore, further studies with this molecule seem to be warranted.

Investigations using synthetic SOD-catalase mimetic, increase in the lifespan of SOD2 nullizygous mice along with recovery from spongiform encephalopathy, and alleviation of mitochondrial defects were observed [149]. These findings lead the authors to hypothesize that the SOD-catalase mimetic could be used as a potential therapy for different neurological diseases related to oxidative stress such as Alzheimer's disease and Parkinson's disease [149].

Studies using type 1 and type 2 diabetic mice models with 60-fold upregulated catalase expression showed amelioration in the functioning of the cardiomyocytes [150]. Cardiomyopathy is related to improper functioning of heart muscles where the muscles become enlarged, thick, or stiff. It can lead to irregular heartbeats or heart failure. Many diabetic patients suffer from cardiomyopathy with structural and functional anomalies of the myocardium without exhibiting concomitant coronary artery disease or hypertension [151].

As already discussed, catalase is interconnected to diabetes mellitus pathogenesis. It has been observed that a 60-fold increase of catalase activity could drastically reduce the usual features of diabetic cardiomyopathy in the mouse model [150]. Due to catalase overexpression, the morphological impairment of mitochondria and the myofibrils of heart tissue were prevented. The impaired cardiac contractility was also inhibited with decrease in the production of reactive oxygen species mediated by high glucose concentrations [150]. So this approach could be an effective therapeutic approach for the treatment of diabetic cardiomyopathy.

## 5. Future Perspective

This review summarizes a relation between catalase and the pathogenesis of some critical diseases such as diabetes, Parkinson's disease, acatalasemia, vitiligo, and Alzheimer's disease. An increase in focus on the role of catalase in the pathogenesis of oxidative stress-related diseases and its therapeutic approach is needed.

Catalase plays a significant role in hydrogen peroxide metabolism as a key regulator [28, 29, 152–154]. Some studies have also shown the involvement of catalase in controlling the concentration of hydrogen peroxide which is also involved in the signaling process [155–158]. Acatalasemia is a rare genetic disorder which is not as destructive as other diseases discussed here, but it could be a mediator in the development of other chronic diseases due to prolonged oxidative stress on the tissues.

We have also discussed the risk of type 2 diabetes mellitus among acatalasemic patients. But more research on the biochemical, molecular, and clinical aspects of the disease is necessary. There are many more questions about acatalasemia and its relation to other diseases which need to be answered. Therefore, further studies are needed to focus on catalase gene mutations and its relationship to acatalasemia and other diseases with decreased catalase activity so that the link can be understood more completely.

The therapeutic approaches using catalase needs more experimental validation so that clinical trials can be initiated. Use of catalase as a medicine or therapy may be a new and broad field of study. Any novel finding about therapeutic uses of catalase will have a huge contribution in medical science. Positive findings can direct towards its possible use for treatment of different oxidative stress-related diseases.

## 6. Conclusion

Catalase is one of the crucial antioxidant enzymes which plays an important role by breaking down hydrogen peroxide and maintaining the cellular redox homeostasis. Diabetes, Alzheimer's disease, Parkinson's disease, etc. are currently becoming common diseases. While there are many factors involved in the pathogenesis of these diseases, several studies from different laboratories have demonstrated that catalase has a relationship with the pathogenesis of these diseases. Research in this area is being carried out by many scientists at different laboratories exploring different aspects of these diseases, but with an ever-increasing aging population, much remains to be achieved. On the other hand, the potential of catalase as a therapeutic drug in the treatment of several oxidative stress-related diseases is not adequate and is still being explored. Additional research is needed to confirm if catalase may be used as a drug in the treatment of various age-related disorders.

## Figures and Tables

**Table 1:** Examples of the various free radicals and other oxidants in the cell [2].

Reactive oxidants	Examples
Reactive oxygen species (ROS)	Superoxide (O_2_^·–^), hydroxyl radical (OH^·^), hydrogen peroxide (H_2_O_2_), alkoxyl radical (RO^·^), lipid alkoxyl (LO^·^), Peroxyl radical (RO2^·^), ozone (O_3_), lipid peroxide (LOOH), singlet oxygen (^1^O_2_), Hydroperoxyl radical (HO_2_^·^)
Reactive chlorine species (RCS)	Hypochlorite ion (OCl^−^), nitryl chloride (NO_2_Cl)
Reactive nitrogen species (RNS)	Nitric oxide (NO^·^), nitrous acid (HNO_2_), Nitrosonium cation (NO^+^), nitrosyl anion (NO^−^), peroxynitrite (ONOO^−^), nitrogen dioxide (NO_2_), alkyl peroxynitrite (ROONO)
Reactive sulfur species (RSS)	Thiyl radical (R-S^·^), perthiyl radical (RSS^·^)

**Table 2:** Physicochemical characteristics of catalase from various sources.

Organisms/organ/organelle	Specific activity (*μ*mol/min/mg)	Optimum temperature (°C)/pH	Inhibitors	*K*_*m*_ value (mM)	Turnover number	Mol. wt.	Reference
*Homo sapiens* erythrocyte (cytoplasm), kidney, and liver (mitochondria, peroxisome)	273800	37°C/6.8-7.5	3-Amino-1-H-1,2,4-triazole	80	-	-	[159, 160]
*Bos taurus* liver	91800	25°C-35°C/6-7.5	3-Amino-1-H-1,2,4-triazole	28.6	-	-	[161–163]
*Oryza sativa*	-	25°C/6-10	Hydrogen peroxide (above 60 mM)	100	80000	234000	[164, 165]
*Vigna mungo* seedling	25700	40°C/7	Cu^2+^, Fe^2+^, EDTA, NaN_3_	16.2	-		[166]
*Escherichia coli*	20700	22°C/6-8	2-Mercaptoethanol	64	16300	337000	[159, 167]
*Saccharomyces cerevisiae*	116100	-	NaCN (35 mM), hydroxylamine	125	-		[159]

**Table 3:** Four different types of Hungarian acatalasemia.

Types	Position of mutation	Types of mutation	Results of mutation	Effect on catalase	References
Type A	Insertion of GA at position 138 in exon 2 occurs which is responsible for the increase of the repeat number from 4 to 5	Frame shift mutation	Creates a TGA codon at position 134	Lacks a histidine residue, an essential amino acid necessary for hydrogen peroxide binding	[168]
Type B	Insertion of G at position 79 of exon 2	Frame shift mutation	Generates a stop codon TGA at position 58	A nonfunctional protein is produced	[169]
Type C	A substitution mutation of G to A at position 5 in intron 7	Splicing mutation	No change in peptide chain	Level of catalase protein expression is decreased	[170, 171]
Type D	Mutation of G to A at position 5 of exon 9	Coding region mutation	Replaces the arginine^345^ residue to histidine or cysteine	Lowering of catalase activity	[172]

## Supplementary Materials

Supplementary Materials (Supplementary Figure 1). In module 1, ACOX1 (peroxisomal acyl coenzyme A oxidase), HSD17B4 (peroxisomal multifunctional enzyme), and HAO1 (hydroxyacid oxidase 1) are involved in the fatty acid oxidation pathway in the peroxisome while the protein DAO (D amino acid oxidase) is involved in the amino acid metabolism pathway in the peroxisome [4–6] (Supplementary Figure 1). All the components of module 1 are involved in different metabolic pathways. The proteins in module 2 are mainly involved in responses against oxidative stress. All the proteins have antioxidant activity except AKT1 (RAC-alpha serine-threonine protein kinase). AKT1 is a serine-threonine protein kinase which is involved in cell survival, metabolism, growth, and angiogenesis. All the proteins of both modules 1 and 2 including CAT have catalytic activity and are located in the lumen of intracellular organelles. SOD2 and AKT1 of module 2 including CAT were involved in the longevity regulating pathway and FOXO signaling pathway in mammals [4–6] (Supplementary Figures 2 and 3). But in multiple other species, SOD1 and SOD3 (superoxide dismutase 3) were also involved along with SOD2, AKT1, and CAT [4–6] (Supplementary Figure 4). Among the reactive species, hydrogen peroxide is freely diffusible and is relatively long-lived. It acts as a weak oxidizing as well as reducing agent; however, it is not very reactive, but it is the progenitor of many other reactive oxygen species (ROS). It has been demonstrated to oxidatively modify glyceraldehyde-3-phosphate dehydrogenase by oxidation of the labile essential thiol groups at the active site of this enzyme [2]. In most cellular injuries, this molecule is known to play an indirect role. One of the most important products is the formation of a more reactive free radical ^·^OH radical in the presence of transition metal ions such as Fe^2+^ by means of the Fenton reaction.
