# Supplementary material for: Role of Catalase in Oxidative Stress- and Age-Associated Degenerative Diseases
Source: Oxid Med Cell Longev. 2019 Nov 11;2019:9613090. doi: 10.1155/2019/9613090 (PMC6885225; doi:10.1155/2019/9613090)
Supplement: Supplementary Materials — (Supplementary Figure 1). In module 1, ACOX1 (peroxisomal acyl coenzyme A oxidase), HSD17B4 (peroxisomal multifunctional enzyme), and HAO1 (hydroxyacid oxidase 1) are involved in the fatty acid oxidation pathway in the peroxisome while the protein DAO (D amino acid oxidase) is involved in the amino acid metabolism pathway in the peroxisome [4–6] (Supplementary Figure 1). All the components of module 1 are involved in different metabolic pathways. The proteins in module 2 are mainly involved in responses against oxidative stress. All the proteins have antioxidant activity except AKT1 (RAC-alpha serine-threonine protein kinase). AKT1 is a serine-threonine protein kinase which is involved in cell survival, metabolism, growth, and angiogenesis. All the proteins of both modules 1 and 2 including CAT have catalytic activity and are located in the lumen of intracellular organelles. SOD2 and AKT1 of module 2 including CAT were involved in the longevity regulating pathway and FOXO signaling pathway in mammals [4–6] (Supplementary Figures 2 and 3). But in multiple other species, SOD1 and SOD3 (superoxide dismutase 3) were also involved along with SOD2, AKT1, and CAT [4–6] (Supplementary Figure 4). Among the reactive species, hydrogen peroxide is freely diffusible and is relatively long-lived. It acts as a weak oxidizing as well as reducing agent; however, it is not very reactive, but it is the progenitor of many other reactive oxygen species (ROS). It has been demonstrated to oxidatively modify glyceraldehyde-3-phosphate dehydrogenase by oxidation of the labile essential thiol groups at the active site of this enzyme [2]. In most cellular injuries, this molecule is known to play an indirect role. One of the most important products is the formation of a more reactive free radical ·OH radical in the presence of transition metal ions such as Fe2+ by means of the Fenton reaction. [file 9613090.f1.docx]

**Supplementary Figures**


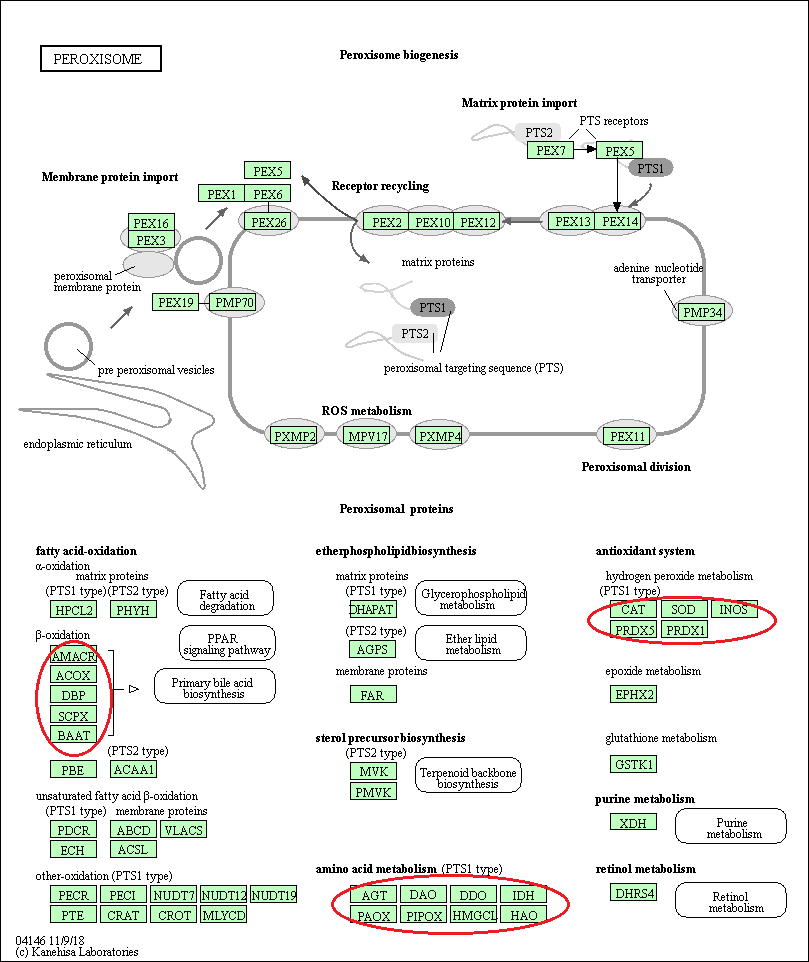


**Supplementary Figure 1**: **Different pathways in the peroxisomes and involvement of different proteins within the pathways.** ACOX (acyl coenzyme A oxidase) is involved in β-oxidation of fatty acids, DAO (D- amino acid oxidase) and HAO (hydroxyacid oxidase) are involved in amino acid metabolism and CAT (catalase), SOD (superoxide dismutase) and PRDX (peroxyredoxin) are involved in hydrogen peroxide metabolism. All the enzymes are located in the peroxisome which is an important organelle involved in redox signaling and lipid homeostasis. Catalase deficiency might increase the hydrogen peroxide concentration inferring with other biochemical and cellular processes.


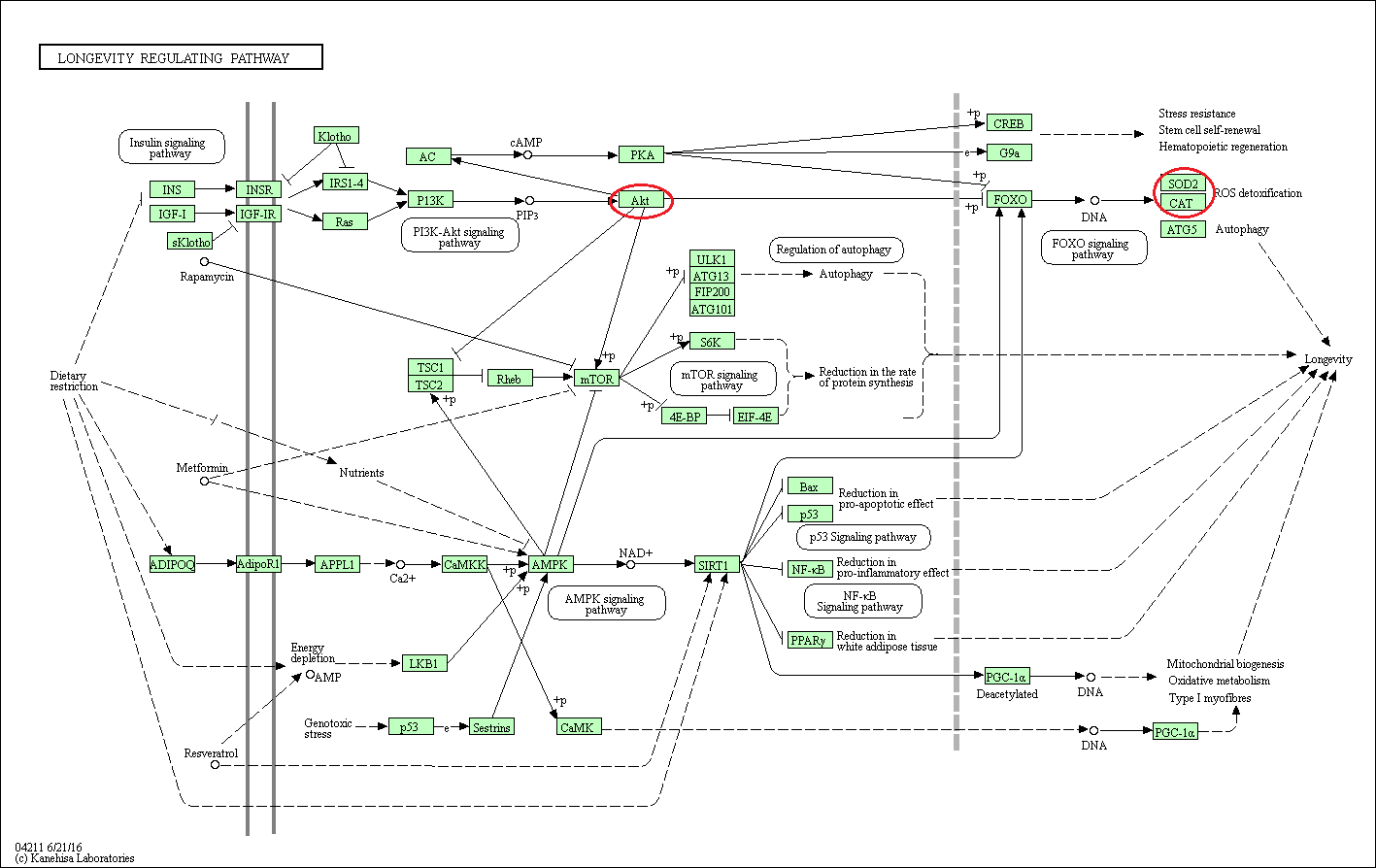


**Supplementary Figure 2:** **Longevity regulating pathways of mammals and the involvement of SOD 2 (superoxide dismutase 2), CAT (catalase) and AKT (alpha serine/threonine protein kinase) within the pathways**. AKT involved in P13K-AKT signaling pathway promotes cell survival, growth and metabolism. Both CAT and SOD 2 are involved in detoxification of ROS. All the signaling pathways are dependent on and connected with each other. Catalase deficiency may create oxidative stress conditions by the resultant increment of hydrogen peroxide concentration, which could affect the other signaling pathways like insulin signaling pathway, sirtuin signaling pathway, AMPK signaling pathway, mTOR signaling pathway. These pathways promote cellular longevity and fitness and, thus, longevity regulation might be affected.


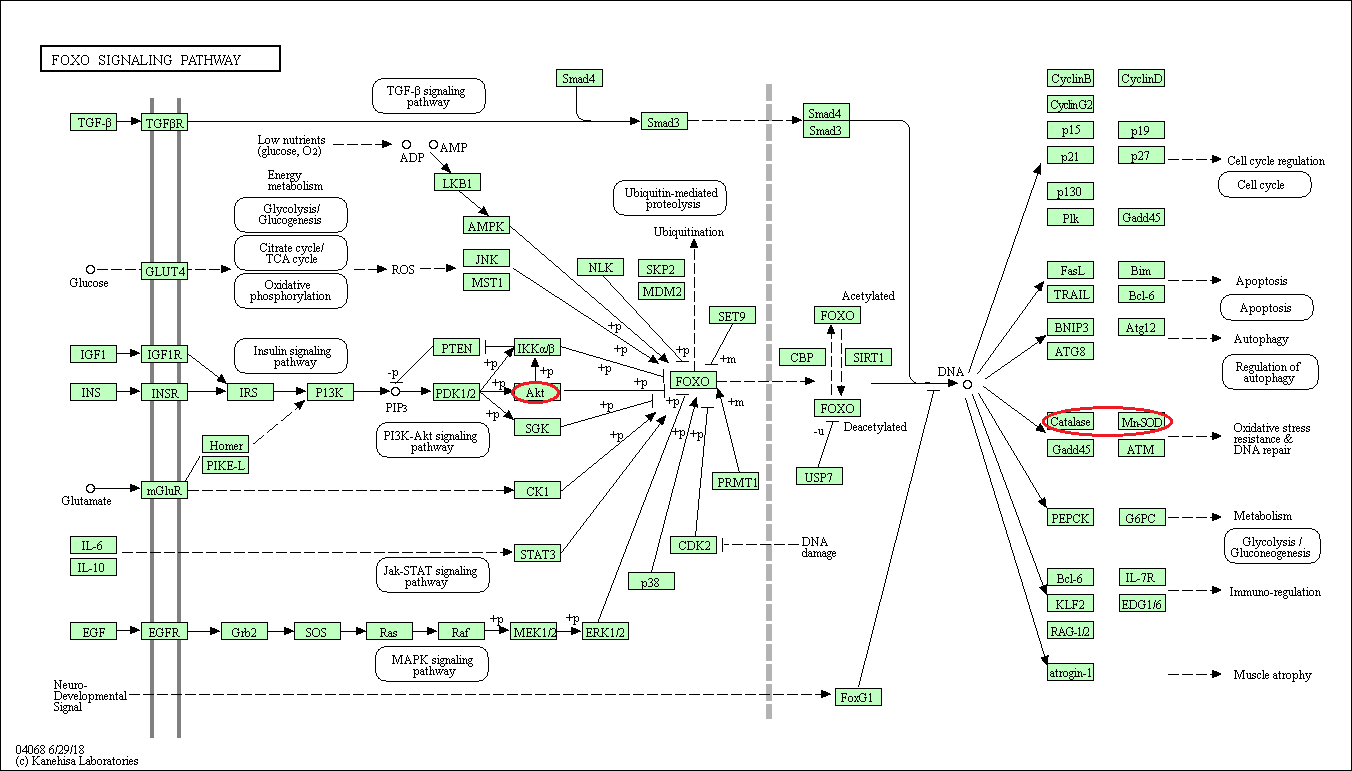


**Supplementary Figure 3**: **FOXO signaling pathway and involvement of SOD 2 (superoxide dismutase 2), CAT (catalase) and AKT (alpha serine/threonine protein kinase) within the pathway.** AKT is involved in P13K-AKT signaling pathway. FOXO signaling pathway along with P13K-AKT signaling pathway are involved in different physiological processes. CAT and SOD 2 are involved in oxidative stress maintenance. The forkhead box O (FOXO) is a group of transcription factors which regulate gene expression of different physiological events within the cell like cell-cycle control, apoptosis, oxidative stress resistance, glucose metabolism and cellular longevity. Catalase deficiency creates an oxidative stress condition due to elevation of hydrogen peroxide concentration. Hydrogen peroxide can regulate FOXO system both positively and negatively through various signaling pathways. The c-Jun-N-terminal kinase (JNK), an important inducer of FOXO activity, can positively regulate the FOXO system [1]. JNK became activated by hydrogen peroxide [1]. When JNK become activated in peroxisome within the cancer cell, an induction of apoptosis occurs through activation of Bax (an apoptosis regulating protein) and negative regulation of Bcl-2 (B-cell lymphoma 2) [2]. In case of negative regulation, hydrogen peroxide-mediated stress condition, Pin1 (peptidyl prolyl isomerase) recognizes the phosphorylated FOXO4 and inhibits transcriptional activity by attenuating its monoubiquitination [3]. The p27^kip1^ expression also inhibits due to inhibition of FOXO4. The inhibition of p27^kip1^ expression leads to the loss of control of cell cycle resulting in carcinogenesis [4].


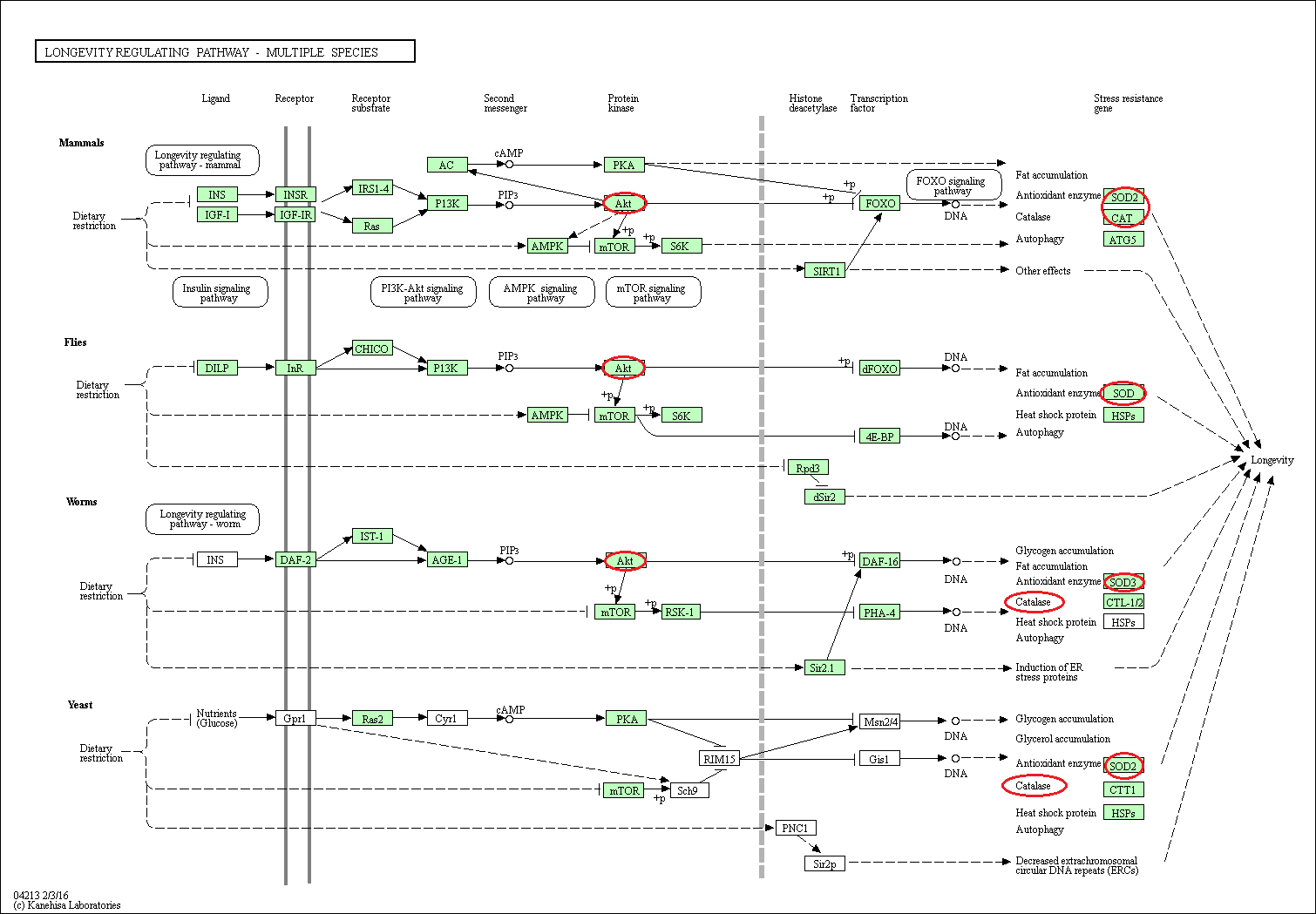


**Supplementary Figure 4**: **Longevity regulating pathways in multiple species and the involvement of SOD 1 (superoxide dismutase 1), SOD 2 (superoxide dismutase 2), SOD 3 (superoxide dismutase 3), CAT (catalase) and AKT (alpha serine/threonine protein kinase) within the pathways.** SOD 2 and CAT along with AKT are involved in longevity regulating pathway in mammals. In case of flies, SOD 1 and SOD 2 are involved along with AKT in the pathway. SOD 3 and catalase are involved along with AKT in longevity regulating pathway in worms. In longevity regulating pathway of yeast, AKT is not involved but both SOD 2 and catalase are involved. The longevity regulating pathway in mammals is discussed in Supporting Figure 2. In flies and worms, it was found that hydrogen peroxide induce the JNK-FOXO signaling pathway involved in the extension of lifespan [5, 6]. But hydrogen peroxide can convert into a more deleterious oxidant, i.e., hydroxyl radical through the Fenton reaction in the presence of transition metal ions. This hydroxyl radical may oxidize different proteins of various signaling pathways and some other protein also. <https://www.kegg.jp/kegg-bin/view_ortholog_table?map=04213>

**References**

1. P. Stortz, “Forkhead homebox type O transcription factors in the responses to oxidative stress,” Antioxidants &Redox Signaling, vol. 14, no. 4, pp. 593-605, 2011.
2. P. Storz, “Mitochondrial ROS-radical detoxification, mediated by protein kinase D, Trend in Cell Biology, vol. 17, no. 1, pp. 13-18, 2007.
3. A. B. Brenkman, P. L. De Keizer, N. J. V. D. Broek et.al., “The peptidyl-isomerase Pin1 regulates p27^kip1^ expression through inhibition of Forkhead box O tumor suppressors,” Cancer Research, vol. 68, no. 18, pp. 7597-7605, 2008.
4. D. Hanahan and R. A. Weinberg, “The hallmarks of cancer,” Cell, vol. 100, no. 1, pp. 57-70, 2000.
5. S.W. Oh, A. Mukhopadhyay, N. Syrzikapa, F. Ziang, R. J. Davis and H. A. Tissrnbaum, “JNK regulates lifespan in Caenorhabditis elegans by modulating nuclear translocation of forkhead transcription factor/DAF-16,” Proceeding of National Academy of Sciences of the United States of America, vol. 102, no. 12, pp. 4494-4499, 2005.
6. M. C. Wang, D. Bohmann and H. Jesper, “JNK signaling confers tolerance to oxidative stress and extends lifespan in Drosophila,” Developmental Cell, vol. 5, no. 5, pp. 811-816, 2003.
